# Retrospective Screening of Anthrax-like Disease Induced by *Bacillus tropicus* str. JMT from Chinese Soft-Shell Turtles in Taiwan

**DOI:** 10.3390/pathogens12050693

**Published:** 2023-05-10

**Authors:** Jia-Ming Tsai, Hsin-Wei Kuo, Winton Cheng

**Affiliations:** 1 Department of Aquaculture, National Pingtung University of Science and Technology, Pingtung 91201, Taiwan; jiamingtsainew@gmail.com; 2 Fish Doctor Veterinary Clinic, Pingtung 94042, Taiwan; 3 General Research Service Center, National Pingtung University of Science and Technology, Pingtung 91201, Taiwan; hwkuo@mail.npust.edu.tw

**Keywords:** *Bacillus tropicus*, *Bacillus cereus*, anthrax-like, protective antigen, anthrax tripartite toxin, *Tryonyx sinensis*

## Abstract

*Bacillus cereus* is ubiquitous in the environment and a well-known causative agent of foodborne disease. Surprisingly, more and more emerging strains of atypical *B. cereus* have been identified and related to severe disease in humans and mammals such as chimpanzees, apes, and bovine. Recently, the atypical *B. cereus* isolates, which mainly derive from North America and Africa, have drawn great attention due to the potential risk of zoonosis. The cluster of *B. cereus* carries several anthrax-like virulent genes that are implicated in lethal disease. However, in non-mammals, the distribution of atypical *B. cereus* is still unknown. In this study, we conducted a retrospective screening of the 32 isolates of *Bacillus* spp. from diseased Chinese soft-shelled turtles from 2016 to 2020. To recognize the causative agent, we used various methods, such as sequencing analysis using PCR-amplification of the 16S rRNA gene, multiplex PCR for discriminating, and colony morphology by following previous studies. Furthermore, the digital DNA-DNA hybridization (dDDH) and average nucleotide identity (ANI) values were calculated, respectively, below the 70 and 96% cutoff to define species boundaries. According to the summarized results, the pathogen is taxonomically classified as *Bacillus tropicus* str. JMT (previous atypical *Bacillus cereus*). Subsequently, analyses such as targeting the unique genes using PCR and visual observation of the bacteria under various staining techniques were implemented in our study. Our findings show that all (32/32, 100%) isolates in this retrospective screening share similar phenotypical properties and carry the protective antigen (PA), edema factor (EF), hyaluronic acid (HA), and exopolysaccharide (Bps) genes on their plasmids. In this study, the results indicate that the geographic distribution and host range of *B. tropicus* were previously underestimated.

## 1. Introduction

The Chinese soft-shell turtle, *Tryonyx sinensis* is well-adapted to aquatic environments and is favored for commercial production because of its high nutritional and medicinal values. In Taiwan, the main pond-breeding area of turtles is located in Kaohsiung and Pingtung counties. In Taiwan, due to the favorable climate, the period for Chinese soft-shell turtle egg-laying lasts about 9 months. The collection and exportation of eggs to China has produced the most important economic income of this industry. Generally, disease occurs when turtles are cultured at high density as a result of overstocked ponds or when turtles are exposed to poor water quality. In recent years, bacterial disease has increased the difficulty of aquaculture management and has led to limited yields, often resulting in significant economic loss [1,2,3]. 

In 2011, several cases of Chinese soft-shell turtle disease originating from *B. cereus* infection were reported for the first time in the pond-cultured farms in Guangdong Province, China [4]. Soon after, similar cases also appeared in Taiwan [5]. In addition to *B. cereus*, *B. thuringiensis* also was isolated from diseased Chinese soft-shell turtles [6]. *B. cereus* group has become a newly emerging bacterial pathogen of Chinese soft-shell turtle in Taiwan. Up to the present date, recurrent epizootic outbreaks have been reported in several pond-cultured farms in Pingtung and Kaohsiung Counties [7]. The emerging epidemic has resulted in severe economic losses to the Chinese soft-shell turtle culture industry in recent years. 

Bacteria within the *Bacillus cereus* group, also known as *B. cereus* sensu lato, are Gram-positive, spore-forming, facultatively anaerobic, rod-shaped bacteria, consisting of at least eight closely related species that are widespread in natural environments [8]. Moreover, nine novel species of the *Bacillus cereus* group were proposed, including *Bacillus tropicus*, *Bacillus paranthracis*, *Bacillus pacificus*, *Bacillus albus*, *Bacillus mobilis*, *Bacillus luti*, *Bacillus proteolyticus*, *Bacillus nitratireducens*, *Bacillus paramycoides* [9]. The most prominent members of this group include *Bacillus anthracis*, *Bacillus cereus*, *Bacillus thuringiensis*, and *Bacillus mycoides*. The group has played a critical role in many aspects of human life, such as terrorism [10], human health [11], agriculture [12], aquaculture [4,6]. 

*B. cereus* is an opportunistic pathogen and a well-known causative agent of foodborne bacterial intoxicants that are known to cause an emetic or diarrheal type of food-associated disease, which is generally self-limiting. Most isolates appear to be harmless [13].

Therefore, it was a dramatic turn when *B. cereus* G9241 was initially isolated from a Louisiana welder in 1994, and subsequently identified as the causative agent of severe anthrax-like pneumonia [14]. In previous study, anthrax lead by *B. anthracis* can induce the systemic effects of edema and severe bacteremia, with bacilli often noticed within blood vessels of the various vital organs [15]. The marked bacteremia are one of the key feature in histopathology [16]. However, a similar disease, such as anthrax, could be triggered by the emerging pathogens.

Since 2004, more and more newly emerging strains of atypical *B. cereus* have been shown to be associated with the anthrax-like disease in humans and mammals such as chimpanzee, apes, and bovine [14,17,18,19,20,21,22]. These atypical *B. cereus* isolates are capable of causing food-borne illnesses and can cause severe, even fatal, infections in non-immunocompromised humans and various animals [23]. As such, *B. cereus* G9241 was thought to differ from typical *B. cereus* isolates by the presence of unique plasmids. The strains harbor two large virulence plasmids, pBCXO1 and pBC210 (or pBCXO2). 

Two components, anthrax toxin and an extracellular capsule, are required for full virulence. The plasmid called pBCXO1, which is the anthrax toxin source of *B. cereus* G9241, shares a high similarity with plasmid pXO1 of *B. anthracis* and consists of the toxin genes *pagA*, *lef*, and *cya* for anthrax toxin components, protective antigen (PA), lethal factor (LF), and edema factor (EF), respectively. 

For extracellular capsules, the hyaluronic acid (HA) component of the capsule is encoded by the *hasACB* operon located on pBCXO1, and the exopolysaccharide (Bps) component is encoded by a nine-gene operon, *bps*X-H, located on plasmid pBC210. This suspected zoonotic disease has attracted significant attention due to the atypical *B. cereus* causing an anthrax-like disease in mammals. Additionally, it is worth mentioning that *Bacillus tropicus* isolated from animals and humans also induced anthrax-like disease in 1941 [24].

On the basis of its highly analogous traits, many members of the genus *Bacillus* could be our suspect, causing the disease and high mortality in the Chinese soft-shell turtle industry. In the study, we estimated the analytical methods of the previous studies [6] and provided an insight into genetic diversity. Genome sequence-based classification was employed to determine taxonomic classification, including orthologous average nucleotide identity (OrthoANI) [25] and digital DNA-DNA hybridization (dDDH) [26]. 

However, as for non-mammals, the distribution of atypical *B. cereus,* which has been implicated in lethal disease, is unknown. In this study, we showed the phenotypical and molecular analysis of *Bacillus* species JMT isolates from Chinese soft-shell turtle with anthrax-like disease. Furthermore, we proposed the taxonomic change for several atypical *B. cereus* isolates.

## 2. Materials and Methods

### 2.1. Case Data Collection in Present Study

#### 2.1.1. Case Profile of Chinese Soft-Shell Turtles

In this study, we collected bacterial isolates, temporarily designated as *Bacillus* species JMT, from diseased Chinese soft-shell turtles. From 2016 to 2020, the epidemic led by *Bacillus* spp. JMT among turtles cultured in Kaohsiung (K) and Pingtung (PT), two geographic districts in southern Taiwan, caused severe mortality in up to 100% of infected animal. A total of 32 cases infected by *Bacillus* spp. JMT were provided by breeders via purposive sampling in the subadult/adult populations of pond-cultured Chinese soft-shell turtle from different ponds. The sources of the *Bacillus* spp. JMT isolates used in the study are listed in Table 1.

#### 2.1.2. Identification of Bacteremia by Histopathology

Isolates JMT107-17 to JMT109-32, were collected for histopathological analysis (Table 1). Vital organs including the heart, lungs, liver, spleen, kidneys, and GastroIntestinal-tract (GI-tract) were sampled and fixed in 10% neutral buffered formalin. Subsequently, organ tissues were processed with standard techniques for dehydration, clearing, embedding, sectioning, staining (both Gram’s stain and hematoxylin and eosin stain), embedding in paraffin, and sectioning. The bacteremia were identified by the presentence of bacilli within blood vessels in various tissues.

### 2.2. Bacteriology Test

#### 2.2.1. Bacterial Isolation, Culture and Collection

Bacteria were isolated from the liver and spleen of Chinese soft-shell turtles by aseptic operation and cultured on trypticase soy agar supplemented with 5% goat blood (BAP) at room temperature (28 °C). The gray–white colonies with β-hemolytic properties can be seen by the naked eye within 24 h. Furthermore, *B. thuringiensis* ATCC 10792 and *B. cereus* ATCC 14579, purchased from Bioresource Collection and Research Center (Hsinchu County, Taiwan), were utilized as the negative positive controls, respectively, to compare with the collected wild strains.

#### 2.2.2. Parasporal Body Visualization with Coomassie Blue Stain

A 0.5 McFarland turbidity standard of the sporulated colony from all isolates was transferred to the microscope slides, and the slides were air-dried before staining. The specimens were stained (0.133% Coomassie Blue stain in 50% acetic acid [27]), rinsed with distilled water, air-dried, and observed by microscope using a 100× oil immersion objective. The appearance of the parasporal body was clearly identified by the presence of abundant dark-blue stains and diamond-shaped objects.

#### 2.2.3. Capsule Visualization with Liu’s Stain and Negative Stain

The spleen from the diseased Chinese soft-shell turtles was imprinted on a microscope slide and dried by air. The tissue stamp smear was completed using Liu’s Solutions including Liu’s A and B solutions (Baso company, Taiwan). Liu’s staining was performed according to the manufacturer’s protocol. Then, the smears were washed with tap water and air-dried prior to microscopic examination. 

The cells from a single colony of each bacterial isolates were scattered into 100 μL of sterilized saline solution and 5 μL of the mixture was pipetted on to a microscope slide and subsequently covered with a coverslip. One drop of India ink (Becton, Dickinson and Company, Franklin Lakes, NJ, USA) was applied on the edge of the cover slide. The presence of encapsulated cells on the wet mount slide was examined microscopically at 100× oil-immersion magnification. 

#### 2.2.4. Plasmid DNA and Genomic DNA Extraction

Plasmid and genomic DNA templates were prepared from the 16 h culture grown on trypticase soy agar, supplemented with 5% sheep blood. Cells were resuspended in 200 μL of sterile MilliQ water. The DNA of each bacterial strain was extracted and purified using Blood and Tissue Genomic DNA Miniprep System and Mini Plus Plasmid DNA Extraction System (Viogene, New Taipei City, Taiwan) according to the manufacturer’s instructions. Finally, purified DNA was used as a template for subsequent PCR reaction and stored at −20 °C until use. Plasmid DNAwaas used for these primer sets, *lef*3/*lef*4, JM*lef*436f/JM*lef*436r, JM*bpsB*208f/JM*bpsB*208r, JM*pagA*318f/JM*pagA*318r, JM*hasA*700f/JM*hasA*700r, JM*cya*882f/JM*cya*882r; genomic DNA was used for the rest of the primer sets (Table 2).

#### 2.2.5. Oligonucleotide Primer Design

16S rRNA and five sets of primers were used to differentiate between specific species among *Bacillus* spp. [29]. PCR assays targeting the *groEL* gene were used to amplify 400 bp of expectant product from *Bacillus* group species. The rest of the sets targeting the *gryB* yielded 253 bp amplicon, 299 bp amplicon, 475 bp amplicon, and 604 bp amplicon for *B. anthracis*, *B. thuringiensis*, *B. cereus*, and *B. mycoides*, respectively. Additionally, we further employed a transcriptional regulator (XRE) as the new biomarker of *B. thuringiensis* [30].

In this study, we designed several new primer sets for detecting unique virulence genes. Specific oligonucleotide primers were designed for hyaluronan synthase and exopolysaccharide biosynthesis protein based on the *hasA* and *bpsB* sequences, respectively (GenBank accession number AAEK01000036 and AAEK01000004); whereas the primers for the PA, EF, and LF were designed from the *pagA*, *cya*, and *lef* sequences, respectively (GenBank accession number CP009592). To confirm the results, another published primer set was undertaken to detect the *lef* gene [31]. The oligonucleotides of all primer sets in this study were synthesized by Tri-I Biotech, Inc. (New Taipei City, Taiwan). The sequences of the primers used in this study are shown in Table 2.

#### 2.2.6. PCR Condition, Gene Cloning and Sequencing 

For each reaction, 50 ng of DNA template and 10 pmole of each primer was added to AccuPower PCR Premix (Bioneer, Daejeon, Korea), consisting of 250 µM of each of the nucleotides dATP, dCTP, dGTP, and dTTP, PCR buffer (10 mM Tris—HCl, 30 mM KCl, 1.5 mM MgCl_2_), and 1 U of Top DNA polymerase. The reaction was added to a total volume of 20 µL with nuclease-free water. The reaction parameters were an initial denaturation at 94 °C for 5 min, 35 cycles of amplification with denaturation at 94 °C for 30 s, annealing at 55 °C for 30 s, an extension at 72 °C for 60 s, and final extension at 72 °C for 7 min. The PCR conditions could be slightly varied to adapt to each primer set. 

The PCR reaction was performed in a FlexCycler2 Thermal Cyclers (Analytik Jena GmbH, Jena, Germany). Finally, the PCR products were analyzed by electrophoresis in 2% agarose gels stained with DNA-View (BIOTOOLS Co., Ltd., Jupiter, FL, USA) and immediately obrserved with UVP GelSolo (Analytik Jena GmbH, Germany) for analysis.

PCR products amplified by each primer set were cloned into the yT&A vector (Yeastern Biotech, Taipei, Taiwan) according to the manufacturer’s instructions and transformed into *Escherichia coli* DH—5α (Yeastern Biotech, Taipei, Taiwan). The recombinant plasmid DNA from the different clones was extracted separately and sequenced using an ABI3730 automatic sequencer (Tri-I Biotech, Inc., New Taipei City, Taiwan). Both forward and reverse strands were sequenced for each individual. For the purpose of ensuring sequence quality, both strands were sequenced as a crosscheck. Determined sequences were compared with the reference strains in GenBank.

The partial *hasA* fragment (GenBank accession number OP588641), partial *bpsB* fragment (GenBank accession number OP588640), partial *pagA* fragment (GenBank accession number OP583942), and partial *cya* fragment (GenBank accession number OP584757) served as the positive control. In addition, the *lef* positive control was kindly provided by the Institute of Medical Science, Tzu-Chi University, Taiwan [32].

### 2.3. Whole Genome Sequencing 

#### 2.3.1. Oxford Nanopore Technologies

Genomic DNA of the representative isolate (JM105-2) was extracted using Pure extraction QIAGEN Genomic-tip 20/G Kit (QIAGEN GmbH, Hilden, Germany). The gDNA extractions were evaluated for quantity and quality using Qubit (Life Technologies Corporation, Carlsbad, CA, USA), and Q6000B UV-Vis spectrophotometer (Quawell Technology Limited, Carlsbad, CA, USA), following the manufacturer’s instructions. Nanopore DNA Libraries were prepared using 2000 ng of gDNA per sample and preprocessed by Short Read Eliminator Kit (Circulomics Inc., Baltimore, MD, USA) to enhance long-read sequencing read lengths by removing short DNA.

Subsequently, libraries construction followed the native barcoding genomic DNA protocol (EXP-NBD114 and SQK-LSK109) (Oxford Nanopore Technologies, Oxford, UK) for combined FFPE repair and end-prep, ligation of barcodes, and the ligation of sequencing adapters. Sequencing was performed with MinKNOW software (version 21.05.8) on the GridION Mk1 using Nanopore sequencing platform by Oxford Nanopore Technologies (ONT) [33]. Subsequently, basecalling reads (Super-accurate basecalling model), and demultiplexing, adapter trimming was analyzed with Guppy (version 4.3.4) (Oxford Nanopore Technologies). De novo assembly was performed using SPAdes (version 3.14.1) [34]. The whole genome sequence of the representative isolate (JMT105-2) was deposited into GenBank (GenBank accession number CP115303, CP115304).

Finally, genome maps of the chromosome and plasmid of JMT105-2 isolate were drawn by Circos software [35] and CLCbio (CLC bio, Aarhus, Denmark), respectively.

#### 2.3.2. Orthologous Average Nucleotide Identity and Digital DNA-DNA Hybridization

Orthologous average nucleotide identity (OrthoANI) between a pair of genomes was calculated by using CJ Bioscience’s Orthologous Average Nucleotide Identity Tool (OAT) [25]. 

Genome-to-genome distance (GGD) analysis was performed using GGD calculator (GGDC) with the GBDP3.0_BLASTPLUS program provided by German Collection of Microorganisms and Cell Cultures (DSMZ, Braunschweig, Germany) (http://ggdc.dsmz.de, accessed on 1 December 2022) [36]. Distances were calculated using recommended GBDP method, formula *d_4_* [26]. These distances were transformed to values analogous to DNA-DNA hybridization (DDH) using a generalized linear model (GLM) and produced digital DDH (dDDH) values. The cutoff values of ANI and dDDH are, respectively, 95–96% and 70% [25,26]. If the value is above the cut-off point, it is recognized as the same species.

Whole genome sequences of the strains for OrthoANI and dDDH analyses were obtained from the GenBank database (https://www.ncbi.nlm.nih.gov/genome, accessed on 3 December 2022). These accession number included *Bacillus tropicus* JMT105-2 (GenBank accession number CP115303), *Bacillus cereus* G9241 (GenBank accession number NZ_CP009590), *Bacillus cereus* strain 03BB87 (GenBank accession number NZ_CP009941), *Bacillus tropicus* strain EMB20(GenBank accession number NZ_CP078081), *Bacillus cereus* ATCC 14579(GenBank accession number NC_004722), *Bacillus thuringiensis* serovar berliner ATCC 10792 (GenBank accession number NZ_CM000753), *Bacillus anthracis* str. Ames (GenBank accession number NC_003997), *Bacillus mycoides* DSM 2048 (GenBank accession number NZ_CM000742).

## 3. Results

### 3.1. Identification of Bacillus spp. JMT by Morphology and PCR 

At first, the colony of *Bacillus* spp. JMT isolates was a white–grey color with a double zone of β-hemolysis in the absence of the rhizoid growth by macroscopic observation after a 24 h incubation period (Supplementary Figure S1). 

**Figure 1:**
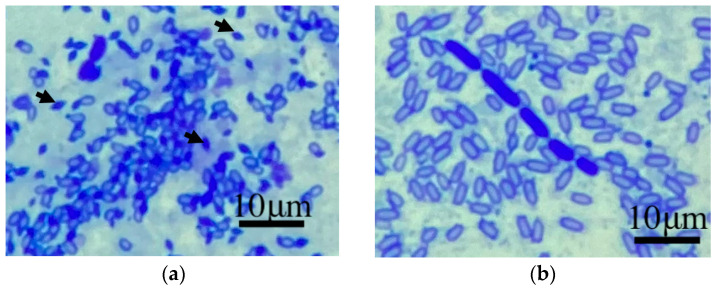
The parasporal bodies of *B. thuringiensis* ATCC 10792 (the arrows) denoted by the presence of dark blue and diamond-shape objects (**a**). No parasporal bodies were present on the specimen of *Bacillus* spp. JMT (**b**).

The parasporal bodies were directly and strikingly marked by the presence of numerous dark blue staining objects. *B. thuringiensis* ATCC 10792 was perfectly demonstrated as a positive control (Figure 1a). Therefore, the dark blue staining bodies were not present in *Bacillus* spp. JMT in the microscopic field (Figure 1b).

The DNA of *Bacillus* spp. JMT isolates were analyzed using the 27f/1492r primer set, and the nucleotide sequence identity among the isolates was 100% after sequencing. The 16S rRNA fragment (1516 bp) of the JMT105-2 isolate was deposited in GenBank (GenBank accession numbers OP592263) and BLAST was conducted on NCBI database. The result we received shared the highest nucleotide sequence identity of 99~100% within three *Bacillus* species: *B. anthracis*, *B. cereus*, and *B. thuringiensis*. Therefore, the published multiplex PCR assay was undertaken to discriminate between the *Bacillus* relatives in the present study by recognizing specific DNA segments. We processed all bacterial isolates in this study, including *B. cereus* ATCC 14579, *B. thuringiensis* ATCC 10792 and all *Bacillus* spp. JMT isolates. One universal and one specific amplicon of each sample in electrophoresis were visible in the PCR reaction of the former two isolates. The rest of the *Bacillus* spp. JMT isolates were unanimous in presenting amplicons and agreed with *B. thuringiensis* ATCC 10792 pattern, which amplified the partial *groEL* fragment (400 bp) and the partial *gyrB* fragment (299 bp) (Supplementary Figure S2).

The transcriptional regulator (XRE) was described as a reliable and capable tool for identifying *B. thuringiensis* in a previous study [30]. The novel biomarker was used to estimate and confirm all bacterial strains. Both the standard strains used as control, *B. cereus ATCC* 14579, *B. thuringiensis* ATCC 10792, showed the expected results. Thus, all *Bacillus* spp. JMT isolates were negative and identical in the use of PCR targeting XRE (Supplementary Figure S2). 

### 3.2. Capability of Capsule Production

**Figure 2:**
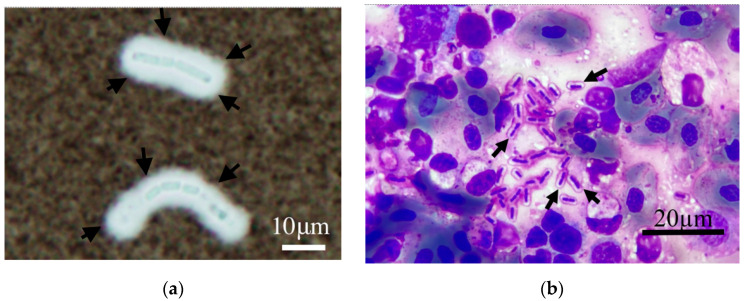
Microscopic images were captured (at 100× oil immersion) of *Bacillus* spp. JMT *was* observed with India ink staining. The unstainable and thick halo surrounding the bacilli on the images was designated the capsule of *Bacillus* spp. JMT (the black arrows) (**a**). Metachromatic color (magenta) and well-demarcated around the bacilli in Liu’s stain (the black arrows) (**b**).

The capsule-producing abilities of 32 *Bacillus* spp. JMT isolates from clinical sources were notably identical (Table 3). We performed a microscopical examination of the wet-mount slides after dropping India ink. One thick and clear layer surrounding the bacilli was observed, as shown in Figure 2a. The capsule was indicated by arrow signs. Negative staining of India ink highlights the location and thickness of the capsule produced by *Bacillus* spp. JMT. In addition, several heterophils with left-shifting were observed on tissue-imprinted smears with Liu’s stain. The capsule was visible as metachromatic color (magenta) and well-demarcated around the bacilli (*Bacillus* spp. JMT), as shown in Figure 2b. In comparison with encapsulated *Bacillus* spp. JMT, only bacilli were observed in *B. cereus* ATCC 14579 strains. 

### 3.3. Anthrax-like Virulence Genes 

#### 3.3.1. PCR Assay

A total of five primer sets were used for screening in this study, specifically targeting each virulence gene of interest. In the capsule-associated genes, *bpsB* and *hasA* were found in all the *Bacillus* spp. JMT (32/32, 100%). 

As for anthrax-like toxin-associated genes, *pagA* and *cya* were present (32/32, 100%) in all *Bacillus* spp. JMT isolates. Unexpectedly, the results of amplifying the partial *lef* gene were negative in all the *Bacillus* spp. JMT isolates using both newly designed and published primer sets. Whether capable of producing the unique capsule or an anthrax-like toxin, all *Bacillus* spp. JMT isolates retained identical properties. All results of the PCR assay are shown in Table 3. The following four nucleotide sequence documents obtained in the current study were submitted to GenBank. These sequences, derived from the JMT105-2 isolate, correspond to the partial *hasA* fragment (GenBank accession number OP588641), partial *bpsB* fragment (GenBank accession number OP588640), partial *pagA* fragment (GenBank accession number OP583942), and partial *cya* fragment (GenBank accession number OP584757).

#### 3.3.2. The Absent Evidence of *lef* Gene via Nanopore Sequencing

A total of 1,442,375 reads (2,441,281,061 bp) were obtained with an average coverage of 236.8. The average read length was 1692.5 bp and the *N_50_* value was 5,257,674 bp. The high-quality short-read and long-read sequences were *De novo* assembled into a complete chromosome and a plasmid using SPAdes Genome Assembler (v. 3.14.1) with default settings. The genome size of JMT105-2 is 5,487,300 bp, containing a chromosome (5,257,674 bp) and a plasmid (229,626 bp) (Supplementary Figure S3). No evidence of known *lef* gene sequences was present in these Nanopore sequencing data. 

### 3.4. Bacteremia

**Figure 3:**
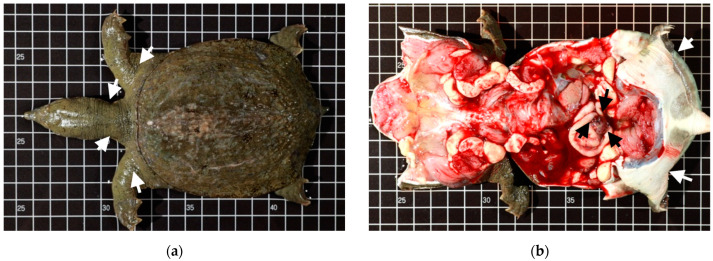
The edema of the diseased Chinese soft-shell turtle can be observed in the neck (dorsal side) (**a**) and limbs (ventral side) (the white arrows) (**b**). The enlarged spleen (the black arrows) was well recognized in the abdominal cavity (**b**).

**Figure 4:**
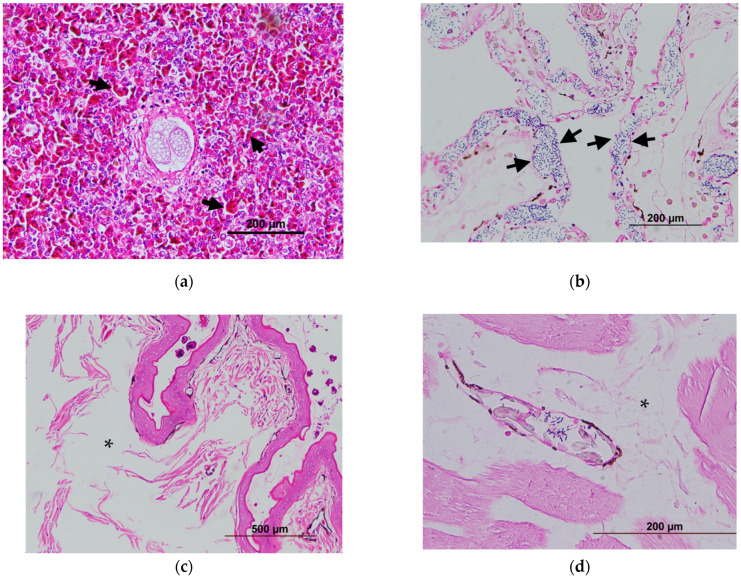
A number of inflammatory cells (mainly heterophil) infiltrating the spleen (the arrows) (**a**). Bacilli denoted by the blue color stained with Gram’s staining in the lung (the arrows) (**b**). Edema (* asterisk) showed (**c**,**d**).

By necropsy examination, the diseased Chinese soft-shell turtle exhibited some morphological changes, such as a generalized edema and darkened and enlarged spleen (Figure 3 and Figure 4). Further, these lesions correspond microscopically to moderate-to-severe edema depending on varying degrees of progress. The vital organs, the lung, liver, spleen, kidney, and GI tract showed diffused pathological changes characterized by inflammatory cells (mainly heterophils) infiltration and bacilli could be noted in blood vessels (Figure 4). Gram-positive bacilli had the same appearance as cytological smears and bacterial cultures. The result of the pathological analysis revealed a highly consistent presence of *Bacillus* spp. JMT-induced bacteremia among 16 clinical cases (16/16, 100%).

### 3.5. OrthoANI and GGD Analyses

Among the eight *Bacillus* spp. strains, the value of seven paired-genome combinations was higher than both thresholds in terms of orthologous average nucleotide identity and digital DNA–DNA hybridization similarities. The calculation recognized as the same species consisted of the following combinations: 1, *Bacillus tropicus* JMT105-2(CP115303) and *Bacillus cereus* G9241(NZ_CP009590.1); 2, *Bacillus tropicus* JMT105-2(CP115303) and *Bacillus cereus* strain 03BB87(NZ_CP009941); 3, *Bacillus cereus* G9241(NZ_CP009590.1) and *Bacillus cereus* strain 03BB87(NZ_CP009941); 4, *Bacillus tropicus* JMT105-2(CP115303) and *Bacillus tropicus* strain EMB20(NZ_CP078081.1); 5, *Bacillus cereus* G9241(NZ_CP009590.1) and *Bacillus tropicus* strain EMB20(NZ_CP078081.1); 6, *Bacillus cereus* strain 03BB87(NZ_CP009941) and *Bacillus tropicus* JMT105-2(CP115303); 7, *Bacillus cereus* ATCC 14579(NC_004722) and *Bacillus thuringiensis* serovar berliner ATCC 10792(NZ_CM000753) (listed in Table 4).

## 4. Discussion

Aquaculture production is essential to the economy and food supply of Taiwan. With the increasing demand for rich protein and high profitability, the aquaculture industry has been motivated to develop in all aspects. The Chinese soft-shell turtle, *Tryonyx sinensis*, is one of the most common and important commercially cultured aquaculture species raised in Taiwan and Southeast Asia. The disease induced by *Bacillus* spp. JMT has had a significant adverse impact on the industry. However, we have known so little about it that we struggle with taxonomic classification. Several methods were developed to deal with this situation. 

### 4.1. JMT Isolates Belong to Bacillus tropicus

The results demonstrated that the etiological agent could be *B. tropicus*. According to phenotypic and molecular analysis, *Bacillus* spp. JMT isolates belong to *B. tropicus* and share the same pattern. In the process of identification, we used various methods to rule out and narrow down possible species. The colony morphology of *Bacillus* spp. JMT indicated a low probability of it being *B. anthracis* or *B. mycoid*. *B. anthacis,* as it is nonhemolytic while *B. mycoid* forms long hair or root-like colonies as rhizoid growth. Thus, the visualization of the parasporal bodies was critical in differentiating *B. thuringiensis* from the *B. cereus* group. The presentation of the crystal protein could be visually discerned by modified Coomassie staining [27]. The assay provided a reliable result in identifying *Bacillus* spp. JMT isolates and led to a definite result; it did not belong to *B. thuringiensis* in terms of phenotypic properties. Nonetheless, *Bacillus* spp. JMT and *B. cereus* 14,579 showed slightly different colony morphologies in terms of texture. The former colony appeared smoother than the latter.

Following molecular analysis, the 16S rRNA fragment was indistinct and not beneficial in distinguishing between species due to its highly conservative DNA sequence. Furthermore, we used a similar strategy as the previous study [6], employing multiplex PCR assay for identification. The result unexpectedly indicated that *Bacillus* spp. JMT belonged to *B. thuringiensis*. After that, the PCR assay targeting the molecular biomarker (XRE) and the multiplex PCR assay results did not match, either. 

The major difference between *B. cereus* and *B. thuringiensis* is that the latter possesses a unique ability to synthesize parasporal crystalline inclusions. Surprisingly, the multiplex PCR primer sets for discriminating in this study apparently failed to identify *Bacillus* species and cannot address *Bacillus* spp. JMT isolates.

Interestingly, the multiplex PCR results of the study by Chen et al. [6] were the same as ours. Moreover, they claimed that *B. thuringiensis* was the pathogen and highly pathogenic to juvenile Chinese soft-shell turtles. Nevertheless, *Bacillus* spp. JMT did not possess the essential ability to produce *B. thuringiensis* crystals in our study.

The conflict between the multiplex PCR results and crystal production ability of the assay requires further high-resolution analysis. Genome-sequence-based analyses provided new insight into the similarity between *Bacillus* species. The digital DNA–DNA hybridization and orthologous average nucleotide identity consequently became a standard technique for the circumscription of bacterial species; we can obtain relatively reliable information to determine genomic similarities. An advantage of these genome-based methods is that they often form sharp, clear clusters of strains compared to those only circumscribed by phenotypic traits [27]. With in silico analyses, the valid published atypical *B. cereus* (strain G9241 and 03BB87) and *Bacillus* spp. JMT105-2 isolate were suggested to be the same species as *B. tropicus* strain EMB20, not *B. cereus* ATCC 14579 or *Bacillus thuringiensis* serovar berliner ATCC 10792. It is worth mentioning that although *B. cereus* and *B*. *thuringiensis* share genomic similarities, they were still considered to be different species [37]. On the basis of the morphology and PCR data, along with the high OrthoANI and dDDH values of *Bacillus* spp. JMT and *B. tropicus* strain EMB20, these isolates from diseased Chinese shell-turtle were assigned as *Bacillus tropicus* JMT. 

As previously mentioned, we solved a confusing problem based on the bacterial isolates collected in the study. The members of the genus *Bacillus* are too close to be distinguished. In previous studies [6,7,38], *B. cereus* and *B. thuringiensis* were considered the etiological agents of Chinese soft-shell turtles. Due to the lack of genome sequence-based analyses, it was difficult to address the taxonomic recognition of these close species-level lineages.

In Taiwan, many Chinese soft-shell turtle *B. cereus*-associated researchers unofficially posit the following theory: the origin of the etiological agent is a biological agricultural pesticide. The typical pond-cultured farms are usually located by country roads and surrounded by agricultural farms in the vicinity. It seems rational to assume that *B. thuringiensis* might be an agent; therefore, it became the most likely suspect. *B. thuringiensis* is deadly to certain herbivorous insects through the production of crystal toxins and is the most frequently used biological pesticide globally.

In this study, all *Bacillus* spp. JMT isolates provided identical results in several tests. Regardless, the etiological agent that triggered the mass death during outbreaks is more likely to be *B. tropicus* than either *B. cereus* or *B. thuringiensis* in Taiwan. According to the summarized results, *Bacillus* spp. JMT is referred to as *Bacillus tropicus* JMT in the following section of the study.

### 4.2. Genome Analysis-Based Reclassification of Atypical Bacillus cereus as Bacillus tropicus

*B. tropicus* was previously recognized as atypical *B. cereus.* In this study, we reclarified the taxonomic status of *B. tropicus* JMT, as well as these atypical *B. cereus* strains.

*B. tropicus* JMT retains several anthrax-like virulence genes and displays different characteristics, such as *B. cereus* G9241 and *B. cereus* 03BB87, not the non-pathogenic *B. tropicus* strain [39,40,41]. In previous studies, these atypical *B. cereus* strains have been widely accepted to overcome host immunity and cause severe symptoms, even death. Since *B. cereus* G9241 has been identified and characterized, the stereotypes of pathogenic *B. cereus* isolates not only originate from food-borne incidents but are also related to severe pulmonary disorders. In addition to being an opportunistic pathogen, it is an etiologic agent of emerging disease, requiring vigilance regarding public health issues. These atypical *B. cereus* strains carry unique virulence toxins encoded on the megaplasmids [14,17,18,20,42,43]. These atypical *B. cereus* plasmids have an analogous function to the pXO1 and pXO2 plasmids of *B. anthracis* and have proven to be sources of pathogenicity [44]. Previous studies have emphasized the diversity between typical and atypical strains in terms of pathogenicity and phenotypic traits. Surprisingly, the genome-sequence-based analyses indicated that we should verify the taxonomic status of all atypical *B. cereus*-associated strains which induce anthrax-like disease.

### 4.3. The Anthrax-like Property of B. tropicus JMT Isolated from Chinese Soft-Shell Turtle

The infectious mechanism of *B. tropicus* JMT isolates could be similar to its relative, *B. anthracis*, which causes a famous zoonotic disease. Anthrax is an acute bacterial disease mainly occurring in wild and domestic herbivores and it occasionally causes acute infection in humans. Like anthrax, atypical *B. cereus* strains were reported to mainly be isolated from mammals. In the present study, we conducted a retrospective screening of the isolates from 2016 to 2020. Our findings provide solid evidence of the presence of *B. tropicus* JMT and its critical pathogenic ability against a reptile: Chinese soft-shell turtle. The capsule could escape from phagocytosis via the host immune system, so this has been considered a virulence factor. For instance, the commonly used laboratory strain *B. anthracis* Sterne harbors plasmid pXO1 only; consequently, its pathogenicity is reduced. Nevertheless, the encapsulated wild *B. anthracis* strain can inhibit both complement-dependent and complement-independent opsonic phagocytosis. [45]. Our findings demonstrated that the capsule production ability was prevalent among the *B. tropicus* JMT isolates. The thick and unstainable coat around the bacilli was visualized by microscopy, using India ink staining in all isolates. The capsule was also visible on the smear with Liu’s staining, which has been used for clinical aquatic animal diagnosis in Taiwan. Its application as a capsule stain with excellent discrimination is similar to the standard staining method used for *B. anthracis* [46]. This characteristic was in agreement with the PCR results of this study and previous atypical strains.

### 4.4. Anthrax-like Disease

The anthrax-like diseases induced by atypical *B. cereus* were usually severe to lethal in mammals in previous studies. We also observed a similar situation in the reptile, Chinese soft-shell turtle. Since 2016, the emerging pathogen has led to a great epidemic of diseases. Bacteremia was recognizable in all vital organs sampled for pathological diagnosis in this study. Previous studies have suggested that the disease may be related to emetic toxin enterotoxins secreted by *B. cereus* [7]. Nevertheless, the classic symptoms we observed in this study were a similar systemic infectious disease rather than an enteric one.

The causative agent, *B. tropicus* JMT, induced similar severe systemic pathological changes in all sampled specimens in this study. The data showed that the diseased animals in this study would suffer from a severe systemic reaction and the circulatory system would eventually be invaded by *B. tropicus* JMT in the terminal stage. In the aquaculture field, the individuals engage the pathogen, inducing a severe serial immune response in the diseased animals. The diseased individuals usually showed lethargic swimming behavior on water surfaces with twisted necks and rigid legs. Further, they exhibited reduced alertness to the surrounding environment. With the course of disease development, abnormal behaviors were observed, accompanied by prominent reductions in feed intake reduction and general edema of the tissues (Figure 3.).

The various cumulative mortality rate of each farm was dependent on the window of opportunity for appropriate antibiotic treatment at the onset of the outbreak. With the progressively declining health condition, the prognosis of the animals acquiring *B. tropicus* JMT infection became increasingly worse. In later stages of outbreak, the cumulative probability of mortality reached 100%. Therefore, the probability of a widespread disaster is much higher in the absence of medical treatment.

### 4.5. Spore Contamination

The environmental pond was contaminated by the sporulated forms after the disease outbreak. Medical treatment with various antibiotics could be effective on the suffering animals. Unfortunately, the rest of animals in the pond were not exempt from invasion of the disease caused by the spores.

The carnivorous habits of the soft-shell turtles also exacerbate the spread of infectious diseases. For animals in the pond, dying animals and the corpses infected with *B. tropicus* JMT were hotspot sources of infection during the outbreak and threatened healthy individuals with overwhelming exposure; in the cultured environment, they grew and sporulated. Subsequently, the sporulated forms shed and were spread throughout the cultured pond by dying animals. When the external conditions were appropriate, the spores germinated and multiplied, increasing the risk of transmission to other healthy individuals in the same pond. The spores are relatively resistant to heat, dryness, and disinfectants. Consequently, the contamination will last as long as there is no way to resist the disease. As a result, healthy individuals acquired the disease annually from batch to batch in the pond contaminated by the spores of *B. tropicus* JMT.

Atypical *B. cereus* isolates rarely inhabit natural soils in the environment [47]. As there is an increasing number contaminated farming areas in Taiwan, the land used to farm Chinese soft-shell turtles has gradually been lost. This concern is consistent with our clinical observations. Owing to the difficulty of spore clearance, *B. tropicus* JMT imposes an adverse economic impact by dramatically decreasing production. In the meantime, a useful decontamination program is desperately needed to control the challenges we will face in the future.

### 4.6. Absence of lef Gene in PCR Assay and Nanopore Sequencing

The *lef* gene was absent in this study. Five newly designed primer sets and one published primer set for screening virulence toxins were employed to confirm the presence of the megaplasmid. The four primer sets were related to the anthrax tripartite toxin; the rest targeted *bpxB* and *hasA*, corresponding to capsule production. Four of the six primer sets amplified the expected PCR products among all the *B. tropicus* JMT isolates. The PCR results imply that *B. tropicus* JMT carries the plasmid that plays a critical role in pathogenicity. Unexpectedly, neither the newly designed nor published primer sets for targeting *lef* could amplify the PCR product. To the best of our knowledge, the genes of *lef*, *pagA*, and *cya* (anthrax tripartite toxin) are encoded on the same plasmid in atypical *B. cereus* and *B. anthracis*. Sequence mutations or incomplete plasmids in *B. tropicus* JMT probably result in amplification failure. The phenomenon of carrying a partial plasmid occurred in *B. cereus* FL2013 [48].

Furthermore, whole-genome sequencing is a powerful tool to provide a high-resolution view of the entire genome. The data of the de novo assembled genome showed no evidence of the segment of *lef* gene. The preliminary result in this study reveals the absence of the *lef* gene.

### 4.7. Remarkable Capsule-Production Capacity of B. tropicus JMT

*B. tropicus* JMT can synthesize astonishingly thick capsules. Both staining visualization methods demonstrated that *B. tropicus* JMT isolates possess an inherent ability to produce capsules.

As for the capsule production, the PCR results correspond to the above-mentioned phenotypic characteristics of *B. tropicus* JMT. The *hasACB* genes are encoded on the plasmid of *B. anthrax* and atypical *B. cereus*. In contrast to *B. anthracis*, atypical *B. cereus* contains non-mutated *hasACB* genes and is functional for capsule production [49]. The capsule produced by *B. anthracis* is considered to help avoid opsonization and phagocytosis of the host immune system [50,51]. Similarly, the atypical *B. cereus* possess this ability due to their two unique natural gifts. Both the virulence plasmids make the required contributions for synthesizing a unique capsule structured with hyaluronic acid (*hasACB*) and polysaccharide (*bpsX-H*). Thus, they differ from the poly-γ-d-glutamic acid (PGA) capsule produced by *B. anthracis*.

In terms of pathogenicity and the thickness of the produced capsule, hyaluronic acid is thought to be a more influential capsule componant than exopolysaccharide in *B. cereus* G9241 [44]. *B. tropicus* JMT isolates carry both the *hasA* and *bpsB* genes related to the formation of capsules. According to the PCR results, capsule-associated genes were present in all *B. tropicus* JMT isolates, implying that they could possibly provide ancillary pathogenic factors. However, whether they have an analogous effect on capsule production compared to previous atypical *B. cereus* isolates remains unknown.

### 4.8. A Biomarker Tool for Rapid Detecting B. tropicus JMT

The powerful primer sets used in the present study are vital for detecting pathogenic *B. tropicus* JMT isolates. *B. cereus* group members are too similar to be recognized as *B. tropicus* JMT in phenotypic analysis. Despite its high pathogenicity and infectivity against Chinese soft-shell turtle, there is no evidence that any other animals or humans could naturally acquire the disease and no medical cases have been documented to date. The living organisms have varying innate susceptibility to the pathogen. Generally, individual species and genetic background are critical determinants of pathogenicity [52]. Using the One Health basics, we recognize that the health of people, animals, and ecosystems is closely connected. Moreover, the spread of many emerging infectious diseases among various animals is also linked and interdependent. *B. tropicus* JMT carries anthrax-like virulence genes, which inevitably give rise to concerns about zoonotic infectious diseases. A thorough understanding of the host range and geographic distribution of *B. tropicus* JMT is essential for the control and prevention of disease outbreaks. Based on previous studies, the distribution of atypical *B. cereus,* which usually infects mammals, has been restricted to North America and Africa. This study is the first retrospective screening report of anthrax-like virulent genes in *B. tropicus* (atypical *B. cereus*) isolated from a reptile and reveals that the atypical strains are pathogens of not only mammals but also reptiles. There is an imperceptible threat that is expanding to affect human and animal health. Additionally, the geographic distribution and host range of *B. tropicus* were underestimated. Extensive surveillance will allow us to better understand and evaluate the epidemic cross-species risk. Furthermore, it will help raise alertness and develop strategies to prevent potential threats. Likewise, for the uphill challenges faced by the aquaculture industry, precise and rapid identification of the etiologic agent is also increasingly important to recover economic losses. Consequently, a reliable tool is needed to assess potentially contagious hazards.

The amplification of each specific virulence gene by PCR can be a valuable tool to distinguish and identify pathogenic *B. tropicus* JMT. The powerful PCR assay based on the anthrax-like gene primer sets JM*bps*B208f/JM*bpsB*208r, JM*pagA*318f/JM*pagA*318r, JM*hasA*700f/JM*hasA*700r, and JM*cya*882f/JM*cya*882r displayed a much stronger ability to identify *B. tropicus* JMT isolates, with the highest detection rate (32/32 = 100%). These results help to confirm that this method is likely to be a viable candidate for screening the causative agents.

## 5. Conclusions

In this study, the bacterial isolates collected from Chinese soft-shell turtles cultured in southern Taiwan during the epizootics of 2016–2020 were *B. tropicus* str. JMT in terms of taxonomic classification. They harbor anthrax-like virulence genes, as shown using a PCR assay (32/32 = 100%). Furthermore, genome sequence-based analyses with high resolution can successfully unveil the lurking emerging pathogen in turtle aquaculture.

Our findings demonstrate that the geographic distribution of atypical *B. cereus* is not only in North America and Africa, but also in Asia. Consequently, the clinical observations imply that *B. tropicus* JMT is a frank pathogen against its host, Chinese soft-shell turtle. Lastly, further studies are needed to elucidate *B. tropicus* JMT’s pathogenic ability against other animals.

## Figures and Tables

**Table 1:** Background information of *Bacillus* spp. JMT isolates were analyzed in this study.

Time	Case (Isolate) ID.	Location	Turtle Age	Cumulative Mortality	Number of Samples	Fixed Tissues ^a^
April 2016	JMT105-1	Changzhi Township (PT)	adult	-	5	−
May 2016	JMT105-2	Ligang Township (PT)	subadult	-	8	−
March 2016	JMT105-3	Ligang Township (PT)	adult	-	4	−
April 2016	JMT105-4	Jiuru Township (PT)	adult	-	3	−
April 2016	JMT105-5	Meinong District (K)	adult	-	7	−
April 2016	JMT105-6	Jiuru Township (PT)	subadult	-	3	−
May 2016	JMT105-7	Yanpu Township (PT)	subadult	-	5	−
May 2017	JMT106-8	Jiuru Township (PT)	subadult	800/1000 (80%)	3	−
March 2017	JMT106-9	Changzhi Township (PT)	adult	300/1200 (25%)	3	−
March 2017	JMT106-10	Ligang Township (PT)	subadult	450/1000 (45%)	2	−
June 2017	JMT106-11	Changzhi Township (PT)	adult	900/1300 (69.2%)	4	−
March 2017	JMT106-12	Jiuru Township (PT)	adult	450/1350 (33.3)	3	−
April 2017	JMT106-13	Ligang Township (PT)	subadult	150/700 (21.4)	4	−
May 2017	JMT106-14	Ligang Township (PT)	subadult	750/1400 (53%)	4	−
April 2018	JMT107-15	Jiuru Township (PT)	adult	650/900 (72.2%)	3	−
June 2018	JMT107-16	Jiuru Township (PT)	subadult	380/1100 (34.5%)	3	−
May 2018	JMT107-17	Yanpu Township (PT)	subadult	230/900 (25.6%)	5	+
March 2018	JMT107-18	Meinong District (K)	subadult	300/1500 (20%)	7	+
March 2018	JMT107-19	Ligang Township (PT)	adult	400/1300 (30.8%)	3	+
April 2018	JMT107-20	Ligang Township (PT)	subadult	600/1300 (46.2%)	3	+
May 2018	JMT107-21	Ligang Township (PT)	adult	1200/1200 (100%)	2	+
March 2018	JMT107-22	Changzhi Township (PT)	adult	800/1450 (55.2%)	5	+
May 2019	JMT108-23	Changzhi Township (PT)	subadult	130/1200 (59.1%)	4	+
May 2019	JMT108-24	Ligang Township (PT)	adult	350/1200 (29.2%)	3	+
May 2019	JMT108-25	Ligang Township (PT)	subadult	400/1250 (32%)	3	+
June 2019	JMT108-26	Yanpu Township (PT)	subadult	350/1450 (24.1%)	6	+
March 2019	JMT108-27	Pingtung City (PT)	subadult	500/130 (38.5%)	5	+
March 2019	JMT108-28	Ligang Township (PT)	adult	400/900 (44.4%)	5	+
April 2020	JMT109-29	Yanpu Township (PT)	subadult	700/1250 (56%)	4	+
May 2020	JMT109-30	Ligang Township (PT)	subadult	130/2400 (54.2%)	4	+
May 2020	JMT109-31	Ligang Township (PT)	subadult	2300/2300 (100%)	3	+
June 2020	JMT109-32	Jiuru Township (PT)	adult	900/1300 (69.2%)	3	+

(K) denotes Kaohsiung City. (PT) denotes Pingtung County. (-) denotes no data in the column of Cumulative Mortality. (−) denote no tissues available in the column of fixed tissue. (+) denotes there were tissues for histopathology analysis. ^a^ included the heart, lungs, liver, spleen, kidneys, and GI-tract.

**Table 2:** Primer sets used in the present study.

Species	Primer	Primer Sequence (5′-3′)	Amplicon Size (bp)	Target Gene	Reference
Bacteria	27f	AGAGTTTGATCMTGGCTCAG	~1500	16S rRNA	[28]
	1492r	TACGGYTACCTTGTTACGACTT			
*B. cereus group*	BCGSH-1F	GTGCGAACCCAATGGGTCTTC	400	*groEL*	[29]
	BCGSH-1R	CCTTGTTGTACCACTTGCTC			
*B. anthracis*	BASH-2F	GGTAGATTAGCAGATTGCTCTTCAAAAGA	253	*gyrB*	[29]
	BASH-2R	ACGAGCTTTCTCAATATCAAAATCTCCGC			
*B. thuringiensis*	BTJH-1F	GCTTACCAGGGAAATTGGCAG	299	*gyrB*	[29]
	BTJH-R	ATCAACGTCGGCGTCGG			
*B. cereus*	BCJH-F	TCATGAAGAGCCTGTGTACG	475	*gyrB*	[29]
	BCJH-1R	CGACGTGTCAATTCACGCGC			
*B. mycoides*	BMSH-F	TTTTAAGACTGCTCTAACACGTGTAAT	603	*gyrB*	[29]
	BMSH-R	TTCAATAGCAAAATCCCCACCAAT			
*B. thuringiensis*	XRE1	AAGATATTGCAAGCGGTAAGAT	246	XRE	[30]
	XRE2	GTTTTGTTTCAGCATTCCAGTAA			
*B. anthracis*	*lef*3	CTTTTGCATATTATATCGAGC	385	*lef*	[31]
	*lef*4	GAATCACGAATATCAATTTGTAGC			
*B. cereus* G9241	JM*lef*436f	GCGGTCATGGTGATGTAGGT	436	*lef*	This study
	JM*lef*436r	ACGTTCAGTGCCTTTTCAGT			
	JM*pagA*318f	CTGGGACGGCTCCAATCTAC	318	*pagA*	This study
	JM*pagA*318r	AGCCTGTATCCACCCTCACT			
	JM*cya*882f	TGCACCTGACCATAGAACGG	882	*cya*	This study
	JM*cya*882r	TCCGGTTTCCTCTCAATTCCA			
	JM*hasA*700f	GTAATGGCCACAACTGGTCACGTGAAC	700	*hasA*	This study
	JM*hasA*700r	AACGTGTTCCCCAACCATTAGA			
	JM*bp*sB208f	AAAACAGACCCCCAATCACC	208	*bpsB*	This study
	JM*bpsB*208r	GTTTCCGCAGGTCAGCATTT			

**Table 3:** Laboratory findings of the characteristics of *B. cereus* group in this study.

Bacterial Isolates	Colony Morphology	Microscopical Examination		PCR Assay							
	Rhizoid Growth	Parasporal Body	Capsule Expression	*B. c* ^a^	*B. t* ^a^	XRE	*bpsB*	*hasA*	*pagA*	*cya*	*lef*
*Bacillus* spp. JMT (32)	−	−	32+	−	32+	−	32+	32+	32+	32+	−
*B. cereus* ATCC 14579 (1)	−	−	−	1+	−	−	−	−	−	−	−
*B. thuringiensis* ATCC 10792 (1)	−	1+	−	−	1+	1+	−	−	−	−	−

Colony morphology: (+) denotes a positive of rhizoid growth visualization; (−) denote a negative of rhizoid growth visualization. Microscopical examination: (+) denotes a positive of parasporal body and capsule visualization; (−) denote a negative of parasporal body and capsule visualization. PCR assay: (+) and (−) respectively denote a positive and negative amplicon after PCR with different primer sets. *B. c: B. cereus*; *B. t*: *B. thuringiensis*. ^a^ The results were based on multiplex PCR assay in this study.

**Table 4:** The calculation of OrthoANI and dDDH between paired genomes.

	1	2	3	4	5	6	7	8
1		96.64 ^#^	96.56 ^#^	96.83 ^#^	92.06	91.80	94.81	89.68
2	70.20 ^#^		99.98 ^#^	97.63 ^#^	91.97	91.77	94.82	89.65
3	70.10 ^#^	99.80 ^#^		97.59 ^#^	91.91	91.61	94.83	89.66
4	72.20 ^#^	78.40 ^#^	78.30 ^#^		92.14	91.92	95.15 ^#^	89.58
5	46.30	46.10	46.10	46.60		96.80 ^#^	91.71	89.49
6	45.40	45.20	45.00	45.40	71.20 ^#^		91.45	89.58
7	59.00	58.70	58.60	60.90	45.00	44.10		89.54
8	38.70	38.60	38.60	38.50	38.10	38.30	38.20	

^#^ denotes the value is higher than the OrthoANI or GGD thresholds. dDDH values are in the bottom-left and OrthoANI values in the top-right. Value unit: %. Strains: 1, *Bacillus tropicus* JMT105-2(CP115303); 2, *Bacillus cereus* G9241(NZ_CP009590); 3, *Bacillus cereus* strain 03BB87(NZ_CP009941); 4, *Bacillus tropicus* strain EMB20(NZ_CP078081); 5, *Bacillus cereus* ATCC 14579(NC_004722); 6, *Bacillus thuringiensis* serovar berliner ATCC 10792(NZ_CM000753); 7, *Bacillus anthracis* str. Ames (NC_003997); 8, *Bacillus mycoides* DSM 2048 (NZ_CM000742).

## Data Availability

Not applicable.

## Supplementary Materials

The following supporting information can be downloaded at: https://www.mdpi.com/article/10.3390/pathogens12050693/s1. Figure S1. The colony morphology of *Bacillus tropicus* JMT; Figure S2. Gels of PCR assays in this study; Figure S3. Genome map of JMT105-2 isolate contains a chromosome and a plasmid.
