# Supplementary material for: Retrospective Screening of Anthrax-like Disease Induced by Bacillus tropicus str. JMT from Chinese Soft-Shell Turtles in Taiwan
Source: Pathogens. 2023 May 10;12(5):693. doi: 10.3390/pathogens12050693 (PMC10221617; doi:10.3390/pathogens12050693)
Supplement: Supplementary file 1 [file pathogens-12-00693-s001.zip › pathogens-2194391-supplementary.pdf]

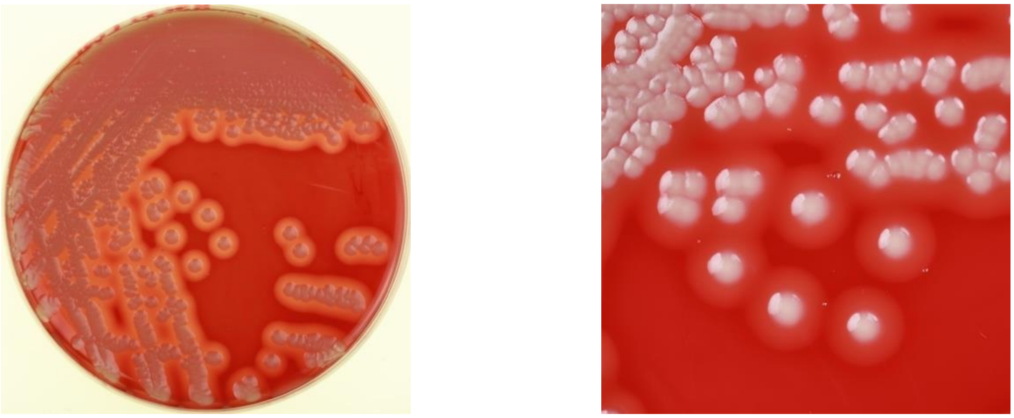

(a) (b)

Figure S1. The colony morphology of *Bacillus tropicus* JMT. The colony on the plate shows white-grey color with a double zone of β-hemolysis in the absence of the rhizoid growth.

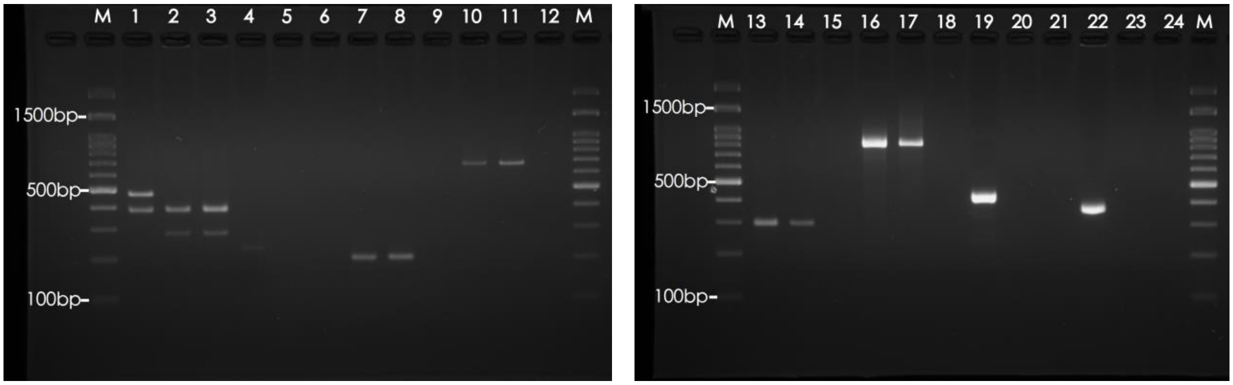

Figure S2. Gels of PCR assays in this study. Using multiplex PCR assays for discriminating among *B. cereus* ATCC 14579 (Lane 1), *B. thuringiensis* ATCC 10792 (Lane 2) and *Bacillus* spp. JMT (Lane 3), the former two were used as control. The expected 400-bp and 299-bp products were obtained together for *Bacillus* spp. JMT with a multiplex PCR primer set targeting *groEL*, and *gyrB* genes, respectively.

XRE1/XRE2 primer set was used for identifying *B. thuringiensis* (Lane 4, 5 and 6). *B. thuringiensis* ATCC 10792(Lane 4) and *B. cereus* ATCC 14579 (Lane 6) were as positive and negative control, respectively. *Bacillus* spp. JMT (Lane 5) was PCR-negative in targeting XRE. *Bacillus* spp. JMT (Lane 8, 11, 14, 17, 20, and 23) were screened by PCR for virulence genes using specific JM*bpsB*208f/JM*bpsB*208r (Lane7, 8 and 9), JM*hasA*700f/JM*hasA*700r (Lane 10, 11 and 12) JM*pagA*318f/JM*pagA*318r (Lane 13,14 and 15), JM*cya*882f/JM*cya*882r (Lane 16, 17 and 18), JM*lef436f*/JM*lef436r* (Lane 19, 20, 21), and *lef3*/*lef4* (Lane 22, 23, 24) primer sets. Lanes 7, 10, 13, 16, 19, and 22 were the positive control, composed of 4 amplicons amplified from each 20ng vector with cloned partial *bpsB*, *hasA*, *pagA*, *cya*, *lef* genes, separately. As well as, *B. cereus* ATCC 14579 was served as the negative control (Lane 9, 12, 15,18,21 and 24). 100bp DNA ladder as the marker (Lane M) were shown on both sides of the gel.

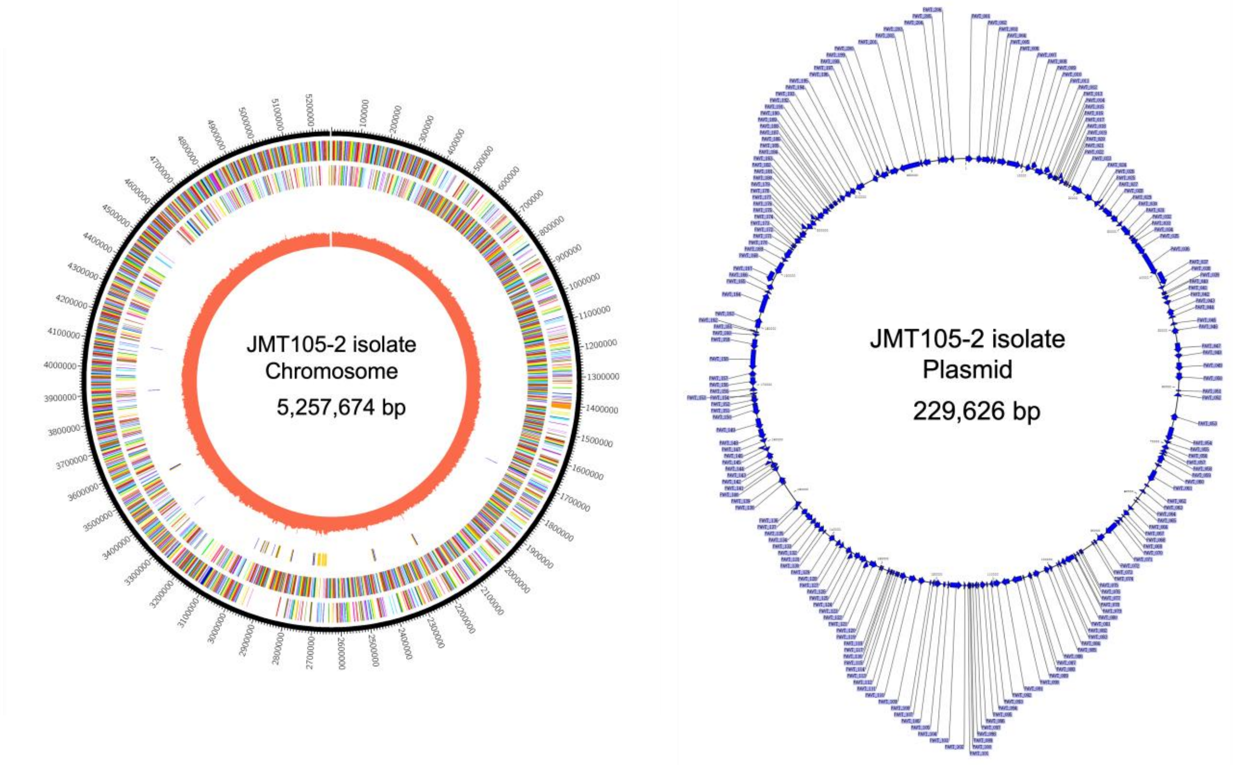

(**a**) (**b**)

Figure S3. Genome map of *B. tropicus* JMT105-2 isolate contains a chromosome and a plasmid.
